# Treatment of Patients With Schizophrenia and Comorbid Chronic Hepatitis With Paliperidone: A Systematic Review

**DOI:** 10.7759/cureus.34234

**Published:** 2023-01-26

**Authors:** Aastha Vats, Athira R Nair, Atithi K Bandhu, Divya Koirala, Manoj R Pallapothu, Maria G Quintana Mariñez, Mohana Chakkera, Niriksha Ravi, Rajita Ramaraju, Ana Francini

**Affiliations:** 1 Psychiatry and Neuroscience, California Institute of Behavioral Neurosciences & Psychology, Fairfield, USA; 2 Research, California Institute of Behavioral Neurosciences & Psychology, Fairfield, USA; 3 Medical Intern, Pontiac General Hospital, Pontiac, USA; 4 Internal Medicine, Tribhuvan University Institute of Medicine, Kathmandu, NPL; 5 Internal Medicine, California Institute of Behavioral Neurosciences & Psychology, Fairfield, USA; 6 Medical School, Pontiac General Hospital, Pontiac, USA; 7 Pediatrics, California Institute of Behavioral Neurosciences & Psychology, Fairfield, USA; 8 Internal Medicine and Neurology, California Institute of Behavioral Neurosciences & Psychology, Fairfield, USA; 9 Psychiatry and Behavioral Sciences, California Institute of Behavioral Neurosciences & Psychology, Fairfield, USA

**Keywords:** diagnostic efficacy, antipsychotics, schizophrenia, hepatitis, paliperidone

## Abstract

Schizophrenia is a severe mental disorder and antipsychotics are drugs usually used to treat this condition. Chronic hepatitis is a condition that can significantly impair hepatic functions. Most antipsychotics are metabolized by the liver, except for paliperidone, which undergoes the least amount of hepatic metabolism. This systematic review was conducted to investigate paliperidone's effectiveness and safety in patients with schizophrenia and concurrent chronic hepatitis. A detailed search using two databases, PubMed and Google Scholar, was done from June 2022 to July 2022. The PubMed search yielded 443 results and three more results were identified from Google Scholar. After a thorough screening, seven results pertinent to our study were taken into consideration for this review. All of the studies suggested that paliperidone is a safe and effective drug for the treatment of schizophrenia and since it does not undergo major hepatic metabolism and has no drug-drug interactions with antiviral drugs given in the treatment of chronic hepatitis, It can be safely used to treat schizophrenia with chronic hepatitis as a comorbid condition.

## Introduction and background

Hepatitis that lasts longer than six months is referred to as chronic hepatitis. The most common causes of chronic hepatitis are hepatitis C, hepatitis B, fatty liver (non-alcoholic steatohepatitis), and alcoholic liver disease. About 60-70% of instances of chronic hepatitis are brought on by the hepatitis C virus, and at least 75% of acute cases progress to chronic hepatitis. About 5-10% of adult cases of hepatitis B, occasionally with hepatitis D coinfection, progress to chronic disease [1]. Schizophrenia is a complex behavioral and cognitive disease with psychotic symptoms. Antipsychotic medication along with psychological therapies, social support, and rehabilitation make up the majority of current treatments [2]. In 2006, paliperidone (9-hydroxy-risperidone), the active metabolite of risperidone, received global approval for the treatment of schizophrenia. Paliperidone has a low likelihood of producing drug-drug interactions since the cytochrome P450 (CYP) enzyme system in the liver only marginally metabolizes it. Although some hepatic metabolism occurs, renal excretion appears to be the main method of paliperidone clearance, with 59% of the oral dose being excreted unaltered in the urine [3]. Paliperidone can be administered orally (extended-release) [3] or as a long-acting injection (LAI) [4].

Nearly two-thirds of newly identified cases of chronic liver disease in the general population are caused by hepatitis C virus (HCV) infection, either alone or in conjunction with alcohol-related liver disease [5]. Around 8.5% to 30% of patients with major mental illness have chronic HCV infection, which is an incidence that is 5-16 times higher than that of the general population [5]. Drug usage, especially injection drug use, is a significant HCV transmission route. In samples of patients with major mental illness, rates of lifetime injectable drug use range from 5% to 35%, compared with 1.4% in the general population. Nearly half of the people with schizophrenia fulfill the criteria for a substance use disorder in their lifetime [5]. Patients with viral hepatitis and schizophrenia exhibit a considerably increased risk of severe hepatic outcomes compared to those without viral hepatitis since atypical antipsychotics can cause drug-induced liver injury [6]. Psychiatrists must take precautions to reduce any potential hepatic harm from certain drugs while treating patients with liver disease. Many commonly used psychiatric drugs (including atypical antipsychotics) are titrated slowly and their doses are reduced as a result of poor hepatic functioning, which causes delays in achieving steady-state concentrations and prolonged elimination half-lives [5].

In the presence of liver disease, medications having substantial first-pass hepatic metabolism can result in high blood levels and side effects at regular dosages and should be carefully titrated [5]. The hepatic metabolism of paliperidone (an atypical antipsychotic) is rather modest [5]. This systematic review aims at finding out the effectiveness and safety of paliperidone (an atypical antipsychotic) in individuals with schizophrenia and chronic hepatitis.

Methods

Protocol

We followed the Preferred reporting items for systematic reviews and meta-analyses (PRISMA) [7] standards for conducting our systematic review.

Eligibility Criteria and Study Selection

This systematic review used the following inclusion criteria: (1) free full texts, (2) all study types, (3) studies conducted on humans, and (4) studies in the English language. The studies excluded were: (1) paid articles or abstracts and (2) animal studies.

Database and Search Strategy

Studies from PubMed were chosen for this systematic review, and the search terms were ("Schizophrenia/drug effects"[Mesh] OR "Schizophrenia/drug therapy"[Mesh] OR "Schizophrenia/metabolism"[Mesh]) AND ("Paliperidone Palmitate"[Mesh])”, “("Hepatitis, Chronic"[Mesh]) AND ("Schizophrenia"[Mesh])”, “Chronic hepatitis and Schizophrenia” and “Schizophrenia and Paliperidone”. We looked for duplicates after the search was finished. An advanced search on Google Scholar was done to yield the desired results and the terms searched were: “Paliperidone AND Chronic hepatitis” and “Schizophrenia AND Paliperidone AND Chronic hepatitis”. The first 10 pages of the advanced search were screened to get the desirable papers. Through the titles and abstracts, the pertinent articles were identified.

Data Extraction and Analysis

The following parameters were collected during the systematic review: research type, the purpose of the study, sample size, the pharmacokinetics of paliperidone, and prevalence of liver disease in schizophrenia patients.

Results

**Figure 1:**
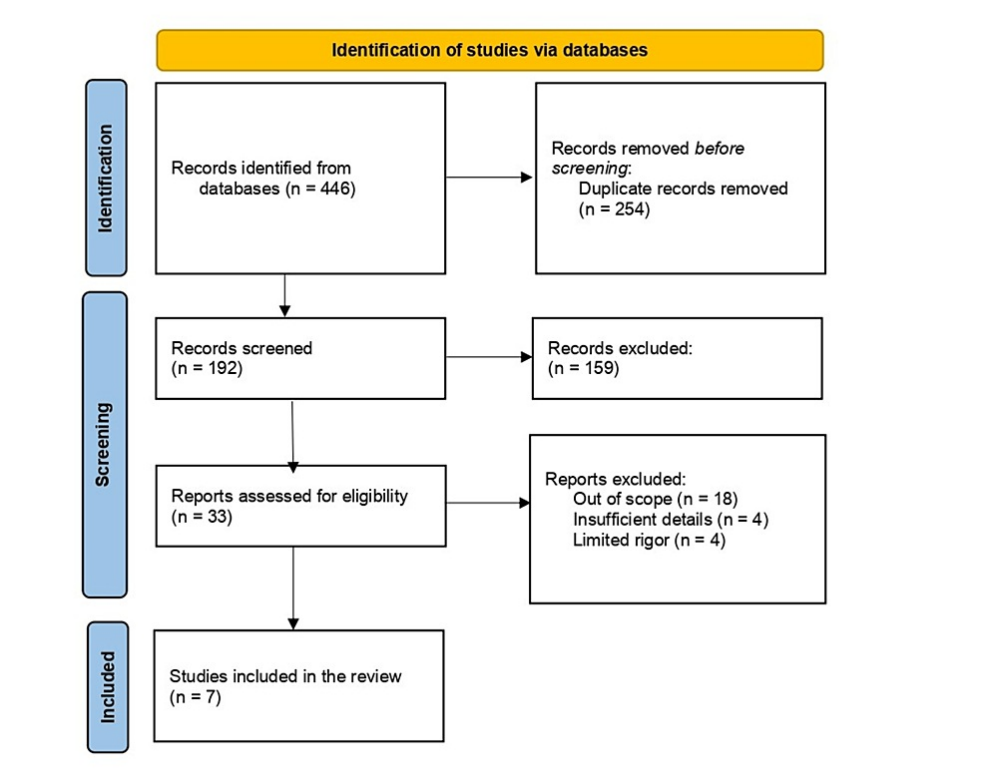
PRISMA flow chart of this systematic review

A total of 446 records were identified after a database search, from which seven studies were included in the review. Details of the selection of research studies are in Figure 1.

Study Characteristics

Out of the seven studies included, there were two case reports, three observational studies, one literature review, and one thematic analysis. Table 1 shows the type of study, method, and findings of each study.

**Table 1:** Studies and their findings

Author	Title of Study	Method	Findings
Viron et al. [5]	Schizophrenia complicated by chronic hepatitis C virus and hepatic encephalopathy	Case report	In populations with severe mental illnesses like schizophrenia, liver disease and its most common underlying causes (alcohol and hepatitis C virus infection) are more prevalent.
Chang et al. [6]	Hepatitis C virus and hepatitis B virus in patients with schizophrenia	Cohort study	Patients who have both viral hepatitis, particularly hepatitis C virus infection, and schizophrenia are at an increased risk of developing serious hepatic conditions such as liver malignancy, failure, or decompensation. Paliperidone use had the lowest risk of a severe hepatic outcome among antipsychotics.
Lv et al. [8]	Antipsychotic drugs and liver Injury	Thematic analysis	Novel antipsychotics primarily indirectly harm the liver by causing metabolic side effects (such as weight gain, obesity, metabolic syndrome, etc.). Monitoring of liver function is still required both before and after treatment.
Carrillo de Albornoz Calahorro et al. [9]	Successful treatment of psychosis induced by interferon-alpha and ribavirin with paliperidone: first case reported	Case report	Unbound plasma concentrations of paliperidone extended-release in patients with moderate hepatic impairment and healthy volunteers were comparable, according to pharmacokinetic studies. As a result, patients with mild or moderate hepatic impairment do not need a dose change.
Hung et al. [10]	Prevalence of hepatitis B and hepatitis C in patients with chronic schizophrenia living in institutions.	Cross-sectional study	The prevalence of hepatitis B virus and hepatitis C virus infections among institutionalized patients with schizophrenia was comparable to that of the general Taiwanese population.
Huckans et al. [11]	The influence of antiviral therapy on psychiatric symptoms among patients with hepatitis C and schizophrenia	Case-control study	Comparable rates of psychiatric symptoms are seen in patients with schizophrenia and hepatitis C virus infection who get antiviral therapy and those who do not, indicating that antiviral therapy does not disproportionately worsen psychiatric symptoms in people with schizophrenia.
Alphs et al. [12]	Onset and persistence of efficacy by symptom domain with long-acting injectable paliperidone palmitate in patients with schizophrenia	Literature review	Paliperidone palmitate (long-acting injection) therapy results in symptomatic improvement in all significant symptom domains as early as Day 4.

Bias Assessment

To assess the bias, we used the following tools: Cochrane bias tool assessment for randomized control trials, New Castles Ottawa tool for other observational studies, JBI check tool for case reports, and SANRA checklist for papers without any clear methods.

## Review

Discussion

This is a systematic review that analyses the effectiveness and safety of an atypical antipsychotic, paliperidone, in patients of schizophrenia suffering from chronic hepatitis.

The current treatment for schizophrenia consists primarily of the administration of antipsychotics [2]. Most antipsychotics undergo hepatic metabolism and are known to cause severe hepatic outcomes [6] and drug-induced liver injury [8]. Paliperidone, a long-acting atypical antipsychotic, is a metabolite of another atypical antipsychotic, risperidone. It is known to have minimal hepatic metabolism [3]. It is a drug that is well tolerated in patients with impaired hepatic function and does not have metabolic drug interactions [9].

Chronic hepatitis leads to a decrease in hepatic metabolism, which leads to a higher concentration of drugs (metabolized by the liver) in the blood. This also leads to an enhancement of the side effects of such drugs. Hence, drugs that undergo the least hepatic metabolism should be used in conditions comorbid with chronic hepatitis. The dose of these drugs should also be titrated accordingly [6]. Most antipsychotics are metabolized in the liver by cytochrome P450 (CYP) [8]. Hence, an antipsychotic that undergoes the least hepatic metabolism and causes the least hepatic side effects should be considered in the treatment of schizophrenia in patients with concurrent hepatitis. Here, we will analyze the effectiveness and safety of one such atypical antipsychotic, paliperidone.

Schizophrenia With Concurrent Hepatitis

The most common cause of disorders characterized by liver inflammation is viral hepatitis, including hepatitis B virus (HBV) and hepatitis C virus (HCV). Schizophrenia, a serious mental illness, has been linked to it more frequently [6]. A study done in Taiwan by Hung et al. [10] showed that chronic schizophrenia patients who were institutionalized did not have higher rates of HBV and HCV than the general population. The hospital where the patients were staying may have averted an increase in HBV and HCV infection in those with schizophrenia due to the comfortable surroundings and medical resources [10].

Chang et al. [6] did a cohort study to evaluate severe hepatic outcomes caused by antipsychotics in patients with schizophrenia with chronic viral hepatitis. The study revealed that HCV-positive schizophrenia patients exhibited a greater incidence of severe hepatic outcomes than HCV-negative patients. It also revealed that patients with viral hepatitis and schizophrenia had a higher risk of developing severe hepatic outcomes such as liver cancer, liver failure, or decompensation, as compared to patients without hepatitis [6]. Systematic reviews have revealed that treatment with most antipsychotics increases the likelihood of hepatic impairment in patients [8].

It is shown that patients with chronic hepatitis C infection who are being treated with antiviral therapy may develop severe mental illnesses. A retrospective study done by Huckans et al. [11] showed that patients with schizophrenia on anti-viral therapy show similar psychiatric symptoms when on therapy and when off therapy. So, schizophrenia should not stop a person from taking anti-viral therapy when infected with the hepatitis C virus [11]. A case report by Carrillo de Albornoz Calahorro et al. [9] evaluated the treatment preferred in patients suffering from psychosis due to interferon and ribavirin therapy (used for viral hepatitis). They concluded that since most antipsychotics are metabolized by the liver, a drug that has the least hepatic metabolism should be preferred so that there is the least possible hepatic damage. They used paliperidone as their drug of choice in such a situation [9].

From the above studies, we could interpret that even though there is no clear-cut correlation between the increase in the prevalence of schizophrenia leading to an increase in the prevalence of chronic hepatitis or vice versa, It is clear that the use of antipsychotics in patients of schizophrenia with concurrent hepatitis can lead to severe hepatic outcomes. We could also interpret that the preferred drug in individuals with schizophrenia and concurrent hepatitis should be a drug that undergoes the least hepatic metabolism.

Use of Paliperidone in Patients With Schizophrenia

Paliperidone has a distinct pharmacological profile that sets it apart from other antipsychotics, including single dose, predominantly renal excretion, low risk of drug-drug interactions, and different pharmacokinetics. Strong evidence shows paliperidone is safe and effective in treating schizophrenia and schizoaffective disorder [3]. Paliperidone can be administered via two routes: oral-extended release [3] or intramuscular long-acting injection [4]. The long-acting injection is paliperidone palmitate which is administered once a month. It is a nanocrystal formulation of paliperidone that is maintained in an aqueous solution [12]. Orally, paliperidone can be administered as paliperidone extended-release which is a special slow-release formulation [3]

Paliperidone has a low likelihood of producing drug-drug interactions since the cytochrome P450 (CYP) enzyme system in the liver only marginally metabolizes it. The only two CYP isoenzymes known to partially degrade paliperidone are CYP 3A4 and 2D6 [3]. In a study done by Jarema et al., it was demonstrated that the efficacy of paliperidone palmitate in the recommended doses resulted in a reduction in the intensity of symptoms and an extension of the duration of the improvement period. According to this study, paliperidone is advised both in the early stages and in the treatment of schizophrenia. Adverse effects of paliperidone use are comparable to that of a placebo, making it more tolerable than other antipsychotics. Due to its high tolerance, individuals tolerated paliperidone palmitate better than they did other antipsychotic medications [4]. Due to paliperidone's equal efficacy to its parent component risperidone, greater safety, and higher patient satisfaction, it may be more advantageous [13]. Also, receiving treatment with paliperidone considerably lowers both the frequency and length of hospital stays. Paliperidone therapy also markedly decreases the number of hospital admissions and relapse-related visits making it more cost-effective [14].

Paliperidone: Safe to Use in Hepatic Impairment?

Paliperidone is only minimally metabolized in the liver and most of it (60%) is eliminated by the kidneys [4]. Since paliperidone undergoes minimal hepatic metabolism, dose reduction in patients with liver illness is not necessary. This is not the case with other antipsychotics [4]. All individuals on standard antipsychotic therapy are more likely to have a hepatic impairment, according to systematic reviews. Second-generation antipsychotics usually cause hepatic damage by causing acute necrotic hepatitis, cholestatic hepatitis with necrosis, hepatocyte damage, nonspecific inflammatory infiltration, and drug-induced reaction syndrome. No relevant studies have found paliperidone to cause hepatotoxicity to such an extent [8]. A study by Carrillo de Albornoz Calahorro et al. [9] states that no dose adjustment is required in patients with mild or moderate hepatic impairment. In the same study, it was also stated that patients with stable active hepatic disease who have schizophrenia or schizoaffective disorder tolerate paliperidone effectively [9]. According to another study by Minwalla et al., it can be difficult to manage senior schizophrenia patients since they often have reduced kidney and liver functions; in such cases, paliperidone has proved to be superior to other antipsychotics as it is less influenced by changes in metabolism [13].

The three recent short-term, randomized, double-blind, placebo-controlled clinical trials conducted by Gahr et al. [3] show that paliperidone seems to be effective, secure, and well-tolerated in adult patients with schizophrenia. The outcomes of the short-term trials were supported by long-term data, which also showed low liability to produce metabolic side effects such as weight gain, hyperglycemia, and lipid dysregulation. As a second-generation antipsychotic, paliperidone appears to be well tolerated and safe to use according to the published research on safety and tolerability thus far. Prolactin elevation and extrapyramidal effects, which both appear to be dose-related, are its main drawbacks. It has been noticed that the incidence of potentially prolactin-associated adverse events does not rise at the same rate as prolactin. There are metabolic changes like weight gain associated with paliperidone but they are less pronounced as compared to other second-generation antipsychotics [3].

After analyzing the above studies, we could interpret that out of all the other second-generation antipsychotics used for the treatment of schizophrenia, paliperidone undergoes the least hepatic metabolism and has the least potential to cause hepatic toxicity or severe hepatic outcomes. It is also shown to be safe and efficacious in the treatment of schizophrenia. It is shown to be well tolerated except for a few side effects that are dose-related, an increase in prolactin levels, and extrapyramidal effects are two major side effects caused. Despite the side effects, according to our analysis and interpretation of the given studies, we can say that paliperidone is a drug that can be used safely and effectively in patients with schizophrenia with concurrent hepatitis.

Limitations

Our systematic review has some drawbacks. Firstly, despite doing a thorough literature search in reputable databases, we cannot rule out the chance of missing a pertinent study because we only included free full-text papers in English. Secondly, there was significant variation in both the reporting of outcomes and the design of the included studies. As a result, there were only a select variety of study designs that met the criteria to be included in each paper. Each article's selection of the study population varied, adding to the variety. Third, there was a paucity of research demonstrating the frequency of chronic hepatitis in schizophrenia patients due to reasons other than viral hepatitis.

Not a single study or trial has been conducted that directly describes the relationship between paliperidone and its adverse effects in a patient with liver disease. Cross-sectional studies should be performed to evaluate paliperidone's safety and effectiveness in patients with liver disease in comparison to other antipsychotics with high liver metabolism. Further studies should be conducted to analyze the interaction between antiviral drugs and paliperidone to establish the safety of paliperidone administration in patients with active hepatitis on antiviral therapy. Despite the described limitations, this systematic review provides useful information on the safety and efficacy of paliperidone in patients with schizophrenia with chronic hepatitis. This is the first analysis, to the best of our knowledge, to describe the effectiveness and safety of paliperidone in patients of schizophrenia suffering from chronic hepatitis.

## Conclusions

The objective of this systematic review was to analyze the safety and efficacy of an atypical antipsychotic, paliperidone, in the treatment of schizophrenia in patients with chronic hepatitis. From the analyzed studies, it can be inferred that there is not enough data that shows the correlation between schizophrenia and hepatitis. It can also be inferred that in the treatment of schizophrenia with comorbid chronic hepatitis, paliperidone can be used to treat the condition effectively and safely. Compared to other antipsychotics, paliperidone undergoes minimal hepatic metabolism and can be safely used in cases of hepatic impairment. Second-generation antipsychotics are known to cause hepatotoxicity, but no relevant studies have shown cases of hepatotoxicity being caused by paliperidone. There is no need to reduce the dose of paliperidone in patients with hepatic derangement. Also, paliperidone has very few drug-drug interactions with the drugs prescribed for chronic viral hepatitis. According to the studies, the use of paliperidone not only reduced the intensity of symptoms in patients with schizophrenia but also extended the duration of the improvement period. Paliperidone is tolerated well by individuals as compared to other commonly used antipsychotics. It can be said that according to the studies analyzed, paliperidone is a safe and efficacious drug that can be prescribed for schizophrenia patients with concomitant hepatic derangement.
